# Room temperature bio-engineered multifunctional carbonates for CO_2_ sequestration and valorization

**DOI:** 10.1038/s41598-023-42905-5

**Published:** 2023-10-05

**Authors:** H. Mohamed, K. Hkiri, N. Botha, K. Cloete, Sh. Azizi, A. A. Q. Ahmed, R. Morad, Th. Motlamane, A. Krief, A. Gibaud, M. Henini, M. Chaker, I. Ahmad, M. Maaza

**Affiliations:** 1 UNESCO-UNISA Africa Chair in Nanosciences-Nanotechnology, College of Graduate Studies, University of South Africa, Muckleneuk Ridge, PO Box 392, Pretoria, South Africa; 2 Nanosciences African Network (NANOAFNET), Materials Research Department, iThemba LABS-National Research Foundation of South Africa, 1 Old Faure Road, Somerset West, PO Box 722, Cape Town, 7129 Western Cape South Africa; 3 College of Graduate Studies, University of South Africa, PRETORIA, South Africa; 4 Chemistry Department (CMI Laboratory), University of Namur, 2 Rue Joseph Grafé, 5000 Namur, Belgium; 5 IMMM, UMR 6283 CNRS, University of Le Maine, Bd O. Messiaen, 72085 Le Mans Cedex 09, France; 6 Physics and Astronomy Department, Nottingham University, Nottingham, NG7 2RD7 UK; 7 INRS-Energie et Matériaux, 1650 Lionel-Boulet, Varennes, QC J3X 1S2 Canada; 8 Experimental Physics Directorate (EPD), National Center for Physics, Islamabad, 44000 Pakistan

**Keywords:** Biophysics, Materials science, Nanoscience and technology

## Abstract

This contribution reports, for the first time, on an entirely green bio-engineering approach for the biosynthesis of single phase crystalline 1-D nano-scaled calcite CaCO_3_. This was validated using H_2_O as the universal solvent and natural extract of *Hyphaene thebaica* fruit as an effective chelating agent. In this room temperature green process, CaCl_2_ and CO_2_ are used as the unique source of Ca and CO_3_ respectively in view of forming nano-scaled CaCO_3_ with a significant shape anisotropy and an elevated surface to volume ratio. In terms of novelty, and relatively to the reported scientific and patented literature in relation to the fabrication of CaCO_3_ by green nano-chemistry, the current cost effective room temperature green process can be singled out as per the following specificities: only water as universal solvent is used, No additional base or acid chemicals for pH control, No additional catalyst, No critical or supercritical CO_2_ usage conditions, Only natural extract of thebaica as a green effective chelating agent through its phytochemicals and proper enzematic compounds, room Temperature processing, atmospheric pressure processing, Nanoscaled size particles, and Nanoparticles with a significant shape anisotropy (1-D like nanoparticles). Beyond and in addition to the validation of the 1-D synthesis aspect, the bio-engineered CaCO_3_ exhibited a wide-ranging functionalities in terms of highly reflecting pigment, an effective nanofertilizer as well as a potential binder in cement industry.

## Introduction

Within the pressing urgency of climate change^1^, de-carbonization processes and related technologies (CO_2_ sequestration, CO_2_ cycling, CO_2_ conversion, …) are extensively investigated in view of reducing the CO_2_ global footprint^2–4^. Cabonates (XCO_3_) in general and Calcium Carbonate (CaCO_3_) specifically could be effectively produced by harnessing atmospheric CO_2_ and converting it into a valuable final product that is of an economical value such as a major cement component^5^, white pigment^6^, green fertilizer^7^ or/and a drug carrier in the health sector^8,9^.

CaCO_3_ makes up almost 4% of the Earth’s crust and has been studied extensively due to its importance in bio-mineralisation in natural systems, including alkalinity generation, and biogeochemical cycling of elements^7,10–12^. Indeed, natural CaCO_3_ which forms through bio-mineralization process^13,14^, has three known natural crystalline forms, vaterite, calcite, and Aragonite, the first one being a metastable poly-crystal^15–18^. However, vaterite has attracted the attention of the scientific community in view of its peculiar optical and biochemical properties^19–22^, which include porosity along with practical relevance and self-assembly synthesis. Henceforth, vaterite particles are likely to be used effectively as biocompatible containers for delivering therapeutic relevant compounds into living cells and tissues^23^. Moreover, CaCO_3_ particles can be successfully employed as templates for the synthesis of polymer hollow capsules made by using a layer-by-layer method, which are also commonly used as drug delivery carriers^24–26^. Several protocols of vaterite synthesis have been reported^19–21^. For example, the process of crystallization of CaCO_3_ occurring through the formation and further transformation of amorphous CaCO_3_ into vaterite and then Calcite has been demonstrated^27^. The 2 other structures of CaCO_3_ i.e. the Aragonite and Calcite forms of CaCO_3_ play a pivotal role in various strategic industries, specifically cement, paint and coatings sectors^12,13,22–25,28^.

From the synthesis viewpoint, and in addition to the established physical^27,29–31^ and chemical^28,32,33^ processes as well as the natural biomimicry for the fabrication of CaCO_3_^34,35^, there is a fast growing methodology consisting of bio-engineering such a compound in a green and sustainable approach. As such, natural systems and natural extracts of plants have been successfully used as effective chelating agents^36–46^.

Within this contribution, an outright green novel bio-engineering approach for the biosynthesis of single phase crystalline nano-scaled CaCO_3_ is validated using H_2_O as the unique solvent and natural extract of *Hyphaene thebaica* fruit as an effective chelating agent. In this room temperature green process, CaCl_2_ and CO_2_ are used as the sole source of Ca and CO_3_ respectively in view of forming nano-scaled CaCO_3_ with a significant shape anisotropy and an elevated porosity.

In terms of novelty and originality, and relatively to the reported scientific and patented literature in relation to the fabrication of CaCO_3_ by green nano-chemistry, the current cost effective room temperature green process can be singled out as per the following specificities:

i. Only water as universal solvent is used,
ii. No additional base or acid chemicals for pH control,
iii. No additional catalyst,
iv. No critical or supercritical CO_2_ usage conditions,
v. Only natural extract of *Hyphaene thebaica* as an effective chelating agent via its phytochemicals and/or enzymatic compounds,
vi. Room temperature processing,
vii. Atmospheric pressure processing,
viii. No extra thermal annealing required,
ix. Nano-scaled size particles
x. Nanoparticles with a significant shape anisotropy (1-D like nanoparticles)

## Experiments, results and discussions

### Synthesis and methodology

Based on previous successful validation of bio-engineering of several simple and binary oxides using various natural oxides^47–58^, *Hyphaene thebaica* L. Mart fruit was specifically selected in this case. This latter was considered in view of the strong chemical chelation effectiveness of its phyto-compounds. Hence and within this contribution, *Hyphaene thebaica* L. was used. The considered *Hyphaene thebaica* L. Mart fruit material was collected from Aswan, Egypt and washed in running distilled H_2_O and kept in shade for drying in South Africa. The dried fruit material was grounded to fine powder and then stored at room temperature in standard zipper bags. For a typical aqueous extraction, 10 g fruit powder was added to 200 ml of dH_2_O and boiled at 80 °C for 2 h on a standard laboratory hot-plate. The resultant mixture was filtered 3 times in view of obtaining a visually clear aqueous extract. The mechanism of Chelation has been discussed and presented in a series of publications^50–58^.

As it will be discussed later (Section "Properties: light scattering and white pigment applications"), It is to be emphasized that the proposed green synthesis of the nano-scaled CaCO_3_ goes through 2 major phases. The first phase consists of the chemical chelation of the CaCl_2_ precursor by the natural extract of *Hyphaene thebaica* fruit (Likely transformation of CaCl_2_ to (CaOH)_2_). The second phase is related to the reaction of CO_2_ bubbled gas with the chelated Ca Cl_2_ i.e. (CaOH)_2_.

Within this study, and in a typical preparation, 3.32 g of Calcium Chloride (CaCl_2_ M = 110.98 g) were added to 100 mL filtered extract solution and stirred for 24 h at room temperature with gentle stirring and after that bubbling with CO_2_. Afterwards, the precipitates from the reaction mixture were collected by allowing them to settle down. Then, the precipitate is collected by centrifugation for 20 min at 4000 rpm. The precipitate was washed thrice in dH_2_O by subsequent centrifugation for 10 min at 4000 rpm, and the precipitates were washed by subsequent centrifugation for 10 min at 4000 rpm. Finally, the washed precipitate was kept for drying at room temperature. The white powder is investigated in its powdered or pelletized forms.

### Materials and characterization

The scanning electron microscopy (SEM) investigations were conducted on a Verios 5 XHR SEM unit. The UV–VIS–NIR diffuse reflectance studies were acquired using an Ocean Optics unit within the spectral range of 250–1100 nm. As a standard reference for reflectance measurements, BaSO_4_ standard was used. With an optical bandgap of ∼ 6 eV, BaSO_4_ exhibits a low absorption extending to the UV band correlated to an elevated UV–VIS and NIR reflectance. The Luminescence measurements were recorded using a fibre-optics linked Ocean Optics system consisting of a UV light-emitting diode source coupled to a high sensitivity QE Pro-FL spectrophotometer. The excitation wavelength was fixed at 240 nm. The X-rays Diffraction measurements were carried out with a Bruker D8 equipped with a copper sealed tube x-ray source producing ${\text{Cu - K}}_{\left\langle 1 \right.}$ radiation at the wavelength of 1.5406 Å from a generator operating at 40 keV and 40 mA (operating at Θ–2Θ configuration). The scanning rate was 0.03° 2Θ per minute from 20° to 85°. The Diffraction patterns were interpreted using MAUD software.

The Thermal Gravimetry Analysis (TGA) and Differential Scanning Calorimetry (DSC) experiments were performed using a Mettler Toledo TGA/DSC 1 Stare System analyser with a horizontal reaction chamber. About 10–20 mg of sample were placed in a cylindrical Al_2_O_3_ crucible. The TGA furnace was constantly purged with Ar gas. The samples were annealed from 25 to 900 °C at a heating rate of 10 °C/min in a stream of N_2_ or Ar “reactive gas” provided directly above the sample with a flow rate of 50 mL min^−1^. A baseline, obtained under the same conditions with an empty Al_2_O_3_ crucible, was subtracted from the measured thermograms. The Luminescence measurements were recorded using a fibre-optics linked Ocean Optics system consisting of a UV light-emitting diode source coupled to a high sensitivity QE Pro-FL spectrophotometer. The excitation wavelength was fixed at 240 nm.

### Morphological investigations

**Figure 1:**
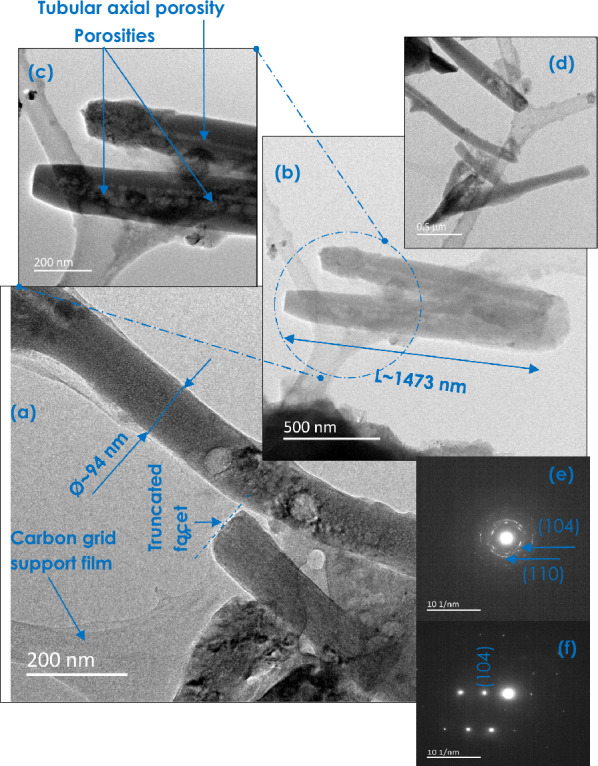
Typical high resolution transmission electron microscopy (HRTEM) and selected area electron diffraction (SAED) of the bio-engineered CaCO_3_.

Figure 1 reports a typical high resolution transmission electron microscopy (HRTEM) and Selected Area Electron Diffraction (SAED) of the bio-engineered CaCO_3_. The particles exhibit nano-scale size and a crystal-clear shape anisotropy in a form of 1-D tubular nanorods with sharp truncated end facets in general (Fig. 1a). Their basal diameter as well as their longitudinal dimension are relatively polydisperse. A typical Basal and longitudinal dimensions of such nanorods are ø_Basal_ ~ 94 nm and D_Long_ ~ 1473 nm. But they can be longer (> 1μm) (Fig. 1d). However, a low magnification observations (Fig. S1a) seem indicating that the CaCO_3_ rods’ basal diameter is relatively disperse size wise. In some cases, they form bundles. Figure S1b reports the normalized basal size distribution. It indicates that the basal dimension of the CaCO_3_ rods varies within the range of 50–800 nm with a relatively large population within the 50–300 nm range.

One should point out that such nanorods seem to be porous. Some nanorods seem, even, exhibiting a hollow longitudinal pores along along their axis (Fig. 1b,c). To sustain the existence of such a porosity, BET investigations were carried out. Figure S2a reports the corresponding profiles. More precisely, Fig. S2b displays the N_2_ adsorption/desorption curves exhibiting the standard isotherm behaviour with a hysteresis-like evolution starting within the vicinity of p/p_0_ ~ 68%. Figure S2a reports a relatively narrow pore distribution of the CaCO_3_ nanorods peaking at about 8.63 nm.

From crystallographic perspective, and as shown in Fig. 1c,d, the biosynthesized CaCO_3_ are not amorphous but crystalline. While the majority of the 1-D tubular CaCO_3_ are polycrystalline (Fig. 1e), some are significantly textured exhibiting an intense spot like electron diffraction pattern (Fig. 1f) as observed previously by Andrews et al.^59^ and Markgraf et al.^60^.

### Elemental analysis and chemical composition

**Figure 2:**
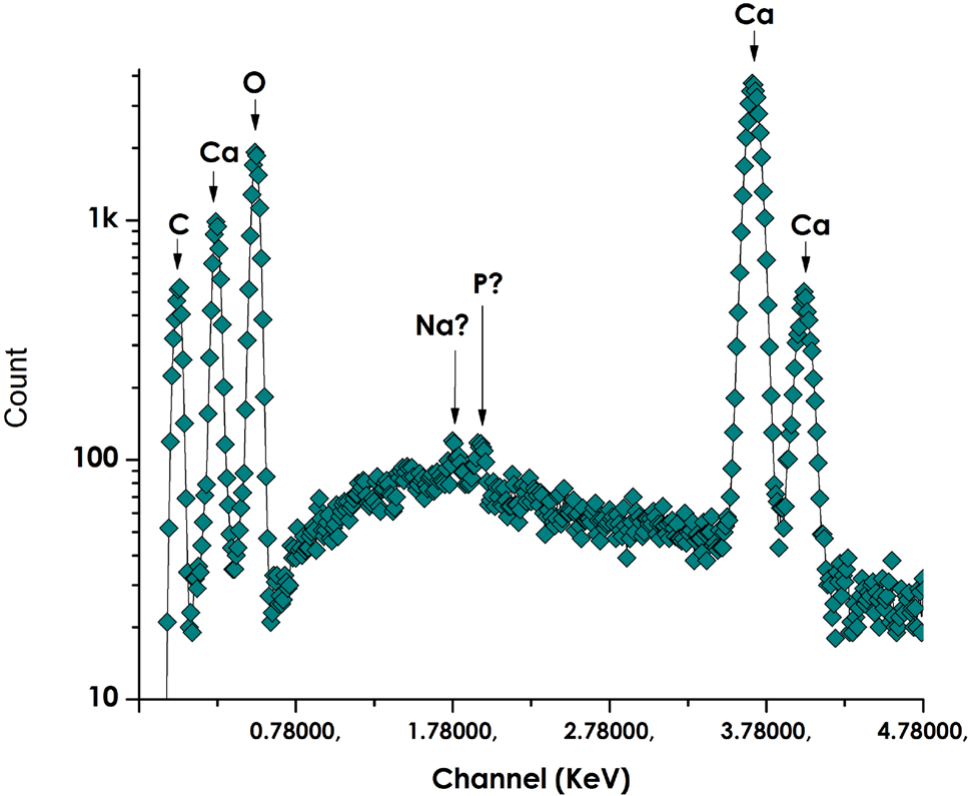
Typical scanning electron spectroscopy (EDS) profile of the bio-engineered nano-scaled CaCO_3_.

Figure 2 displays a typical Scanning Electron Spectroscopy (EDS) profile of the bio-engineered nano-scale CaCO_3_. It reveals 5 major peaks centred at various energy channels of ~ 0.04, ~ 0.27, ~ 0.52, ~ 3.96 and ~ 4.02 keV. According to the EDS database, they are attributed to C, Ca, O, Ca, Ca and Ca respectively (The C peak is attributed to the Carbon coating required for for the EDS investigation). In addition to these intense peaks, and yet they are at the background level, one could observe 2 additional peaks located at ~ 1.78 and ~ 1.94 keV which might be assigned to Na, K and/or P. These later contaminant elements originate likely from the natural extract itself as it was observed in various other oxides bio-synthesized via natural extracts as in the case of bulk CaCO_3_^47–49^. These elements are characterized by a relatively small ionic radius and fast diffusion within any potential channels within the synthesized CaCO_3_.

### Thermogravimetry analysis

**Figure 3:**
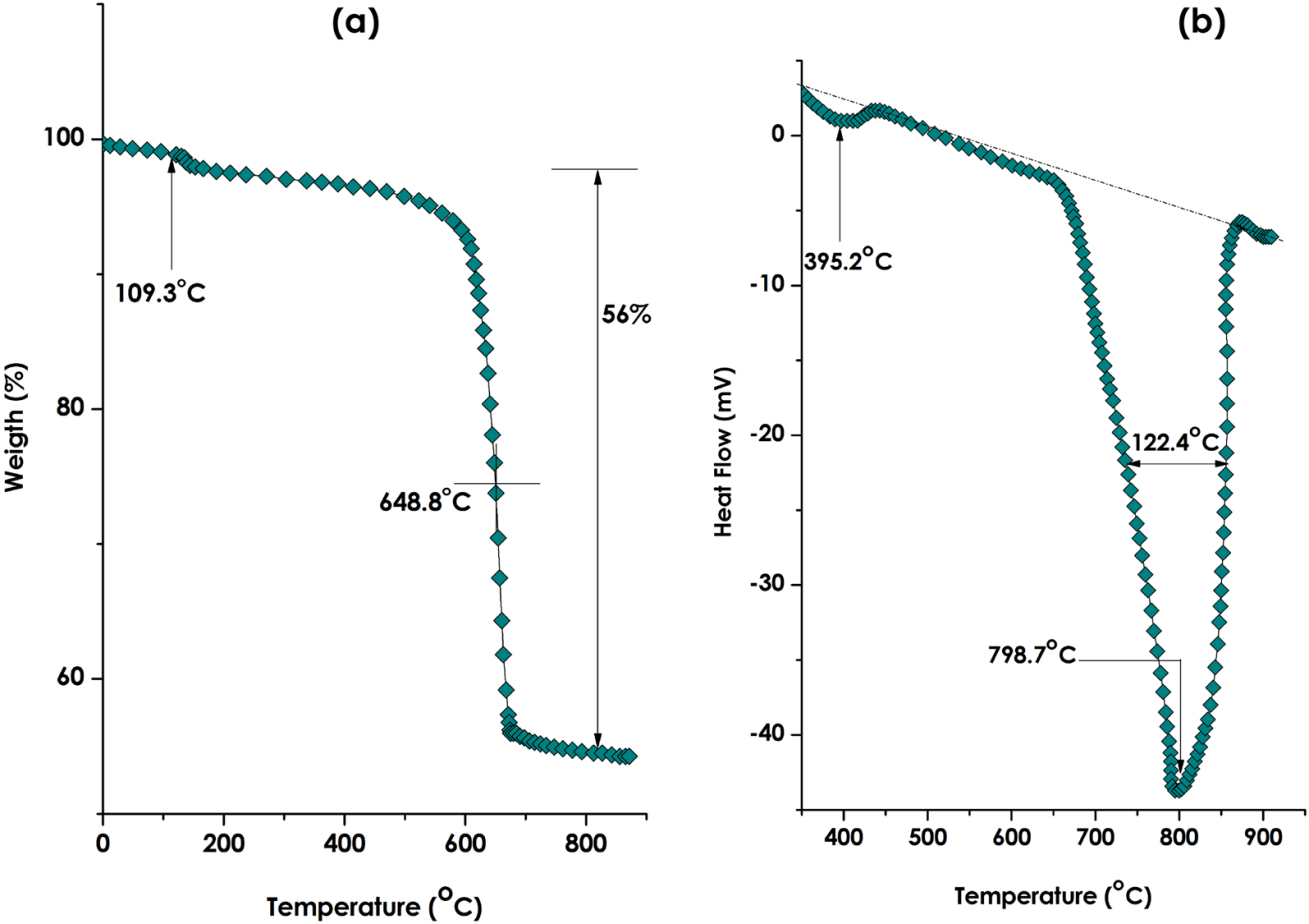
(**a**) Thermo gravimetry analysis (TGA) of the bio-engineered nano-scale CaCO_3_ within the thermal range of 25–850 °C, (**b**) the corresponding differential scanning calorimetry (DSC) profile within the thermal range of 25–900 °C.

Figure 3a reports the Thermo Gravimetry Analysis (TGA) of the bio-engineered nano-scale CaCO_3_ within the thermal range of 25–900 °C. There are, a priori, 2 major decompositions taking place at about ~ 109.3 and ~ 648.8 °C respectively. The first one is likely to be related to H_2_O molecules adsorbed onto the surface, outer or inner interfaces and/or the porosities of the nano-scaled CaCO_3_.The second one, taking place at the vicinity of ~ 648.8 °C, is likely related to the decomposition mechanism of CaCO_3_ corresponding to 56% in weight loss. Following such, CaO crystals and gaseous CO_2_ are formed according to CaCO_3_(s) → CaO(s) + CO_2_(g)^61^.

Figure 3b reports the Differential Scanning Calorimetry (DSC) profile within the thermal range of 25–900 °C. One can distinguish 2 major exothermic phase transitions centred approximately at ~ 395.2 and ~ 798.7 °C. Both phase transitions are not 1st order type considering their width at half maximum which are about ∆T_1/2_ (395.2 °C) ~ 103.7 °C and ∆T_1/2_ (798.7 °C) ~ 122.4 °C. One should point to the absence of the exothermic peak of Ca(OH)_2_ normally taking place around ~ 400 °C. This indicates that the formation of CaCO_3_ is complete without, a priori any trace of the intermediary compound Ca(OH)_2_^61^.

Compared to literature, while the general TGA and DSC evolutions/trends are, grosso-modo, equivalent to that of bulk CaCO_3_ but with a significant shift to lower temperatures. More accurately, the decomposition and phase transition temperature are ~ 648.8 °C instead of ~ 750 °C for Bulk^61^. This is likely due to the high surface to volume ratio of the current nanoscaled CaCO_3_ comparatively to their bulk equivalent.

### Vibrational properties: Raman and Fourier transform infrared spectroscopy investigations

**Figure 4:**
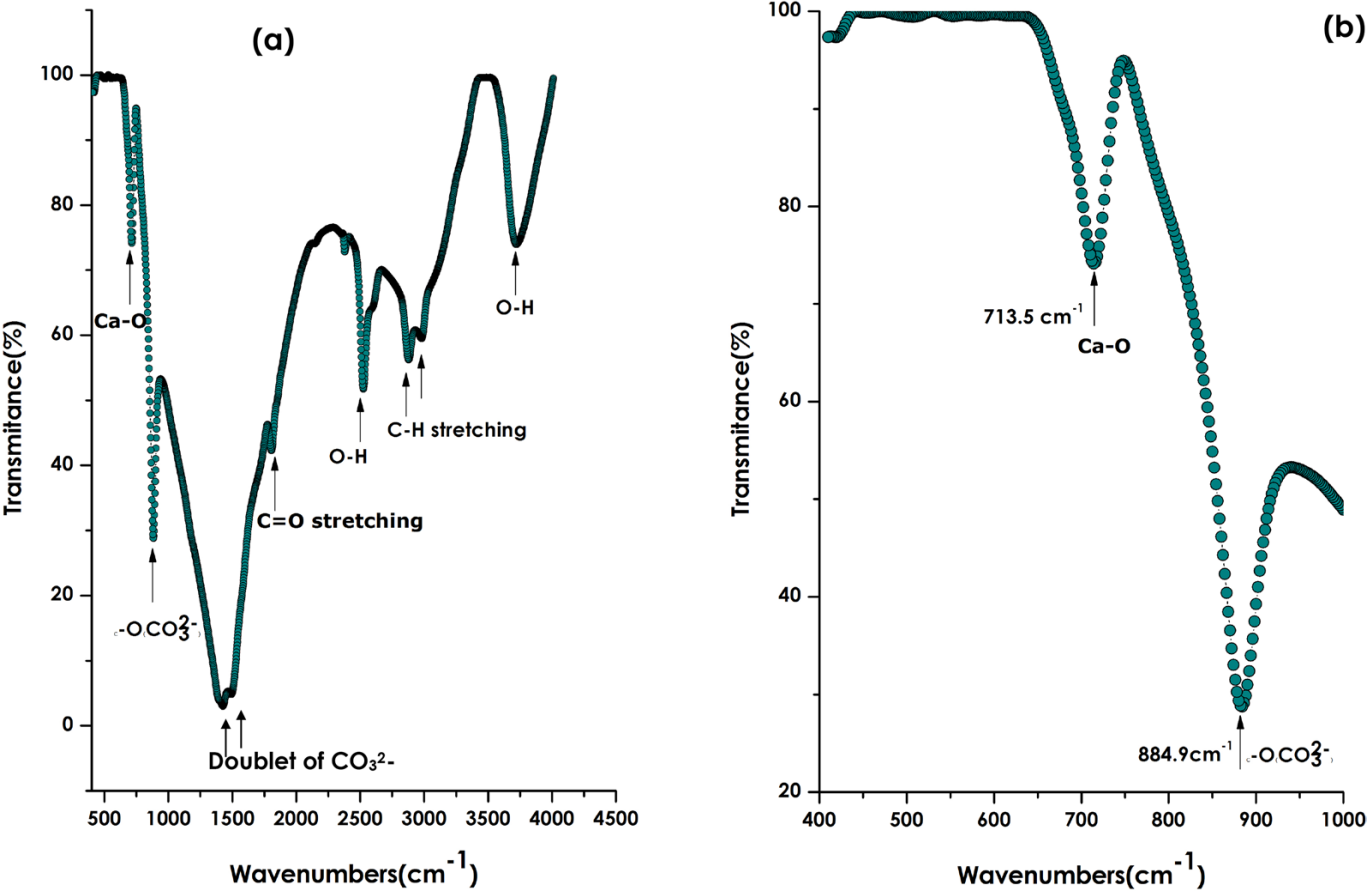
(**a**) Room temperature Fourier Transform Infrared spectroscopy spectrum of the bio-engineered nano-scale CaCO_3_ within the spectral range of 400–4500 cm^−1^, (**b**) zoom on the spectral region of 400–1000 cm^−1^ reporting the characteristic Raman active modes of Calcite CaCO_3_ at 288 cm^−1^ (L_Calcite_) and 161 cm^−1^ (T_Calcite_).

Figure 4a displays the room temperature Fourier Transform Infrared spectroscopy spectrum of the bio-engineered nano-scale CaCO_3_ within the spectral range of 400–4500 cm^−1^. One can distinguish several absorption bands. These later are centered approximately at 3713.5, 2857.8, 2519.7, 1792.5, 1314.5, 866.6, 713.5 cm^−1^. Excluding those centered at 866.6 and 713.5 cm^−1^, the rest which are relatively broad absorptions, are related to O–H modes, C–H streching, O–H modes, C=O stretching and C–H bending vibrational modes. The singular sharp ones positioned at about 866.6 cm^−1^ and 713.5 cm^−1^ are proper to CaCO_3_. More precisely, while the one localized at 866.6 cm^−1^ is a characteristic of the O-CO_3_^2^ vibrational mode in calcite. It is to be highlighted that samples containing CO_3_^2−^ groups, as the case of CaCO_3_, usually exhibit a doublet associated to the v3 vibrational mode close to 1400–1500 cm^−1^ in FTIR. The band with two shoulders clearly visible close to this region are in fact the doublet of CO_3_^2−^^62–64^. The singular absorption at 713.5 cm^−1^ is proper to the Ca–O vibrational mode in calcite and not in aragonite nor vaterite phases as highlighted in the zoom of Fig. 4b^65^. Indeed, and as per the precise investigations of Vagenas et al.^65^, the Ca–O mode’s infrared absorption bands are positioned at 713 cm^−1^ for calcite, 745 cm^−1^ for vaterite, and 700 cm^−1^ for aragonite^65^.

**Figure 5:**
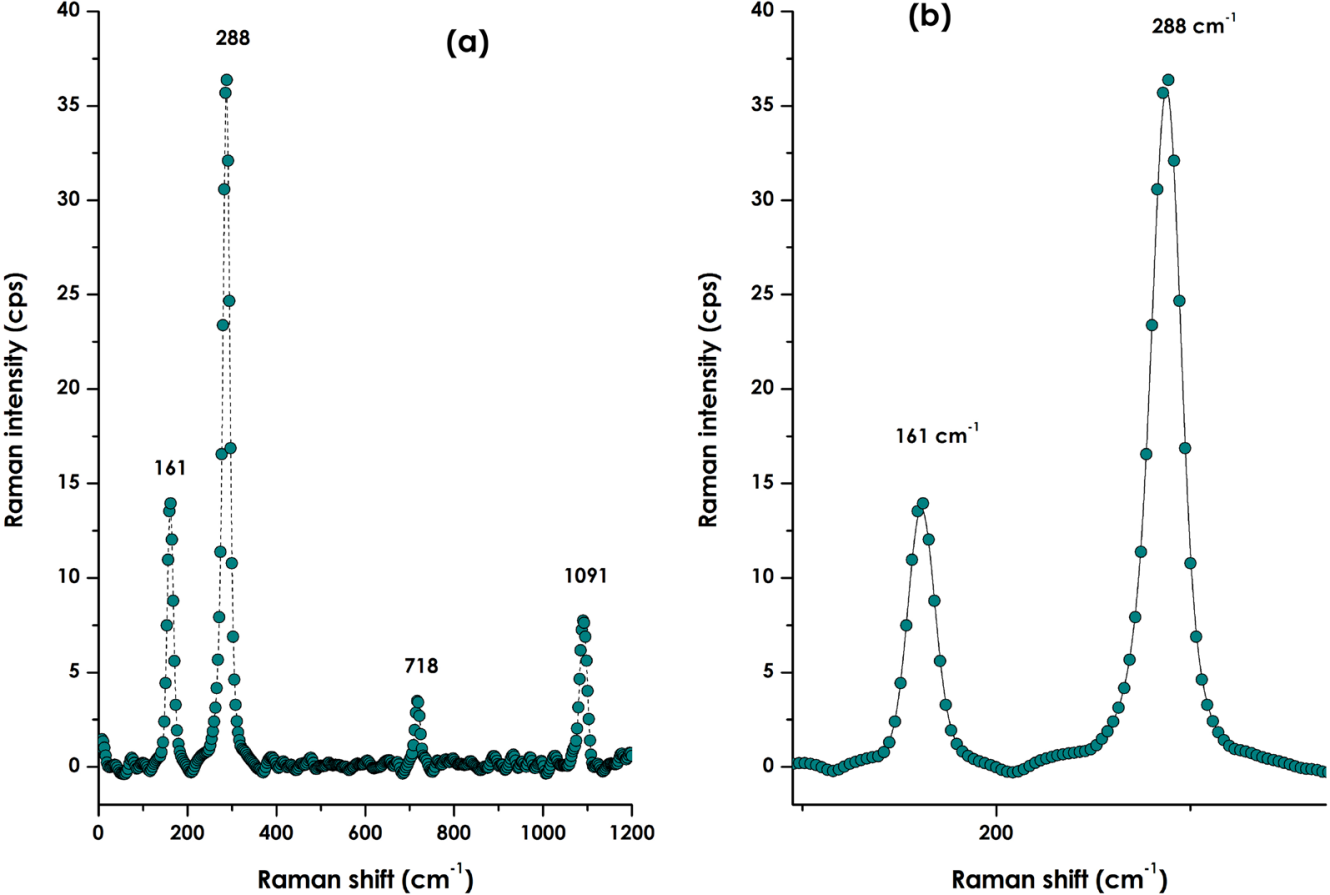
(**a**) Room temperature Raman spectrum of the bio-engineered CaCO_3_ nanoparticles within the range of 0–1200 cm^−1^, (**b**) zoom on the spectral region of 100–370 cm^−1^ reporting the Ca-O characteristic vibrational mode of Calcite CaCO_3_.

Figure 5a reports the room temperature Raman spectrum of the bio-engineered CaCO_3_ nanoparticles within the range of 0–1200 cm^−1^.One can distinguish 4 major peaks centered approximately at the vicinity of ~ 161, ~ 288, ~ 718 and ~ 1091 cm^−1^. While the 161 mode is a Transversal vibrational mode common to both Calcite and Aragonite, the mode located at 288 cm^−1^ is a longitudinal vibration mode characteristic of calcite. More precisely, while the mode at ~ 1091 is an A_1g_, those centered at 161, 288 and 718 cm^−1^ correspond to E_g_ modes of the calcite phase^53^. One should mention however that the rhombohedral primitive cell of calcite contains 2 CaCO_3_ formula units, for a total of 10 atoms; its 27 vibrational modes can be classified according to the irreducible representations of the 3 m point group as follows: Γ_total_ = 1A1g ⊕ 2A1u ⊕ 3A2g ⊕ 3A2u ⊕ 4Eg ⊕ 5Eu. A_1g_ and E_g_ (double degenerate) modes are Raman active, A_2u_ and Eu (double degenerate) are IR active, A_1u_ and A_2g_ are spectroscopically inactive (silent modes).

Last not least, and as highlighted in the zoom of Fig. 5b, one should notice the clear-cut absence of any additional Raman peak between 288 cm^−1^ (L_Calcite_) and 161 cm^−1^ (T_Calcite_) especially the distinctive L_Aragonite_^66^. Consequentially, one could safely conclude that the bio-engineered CaCO_3_ nanoparticles are of pure single phase calcite nature.

### Defects investigations: photoluminescence studies

**Figure 6:**
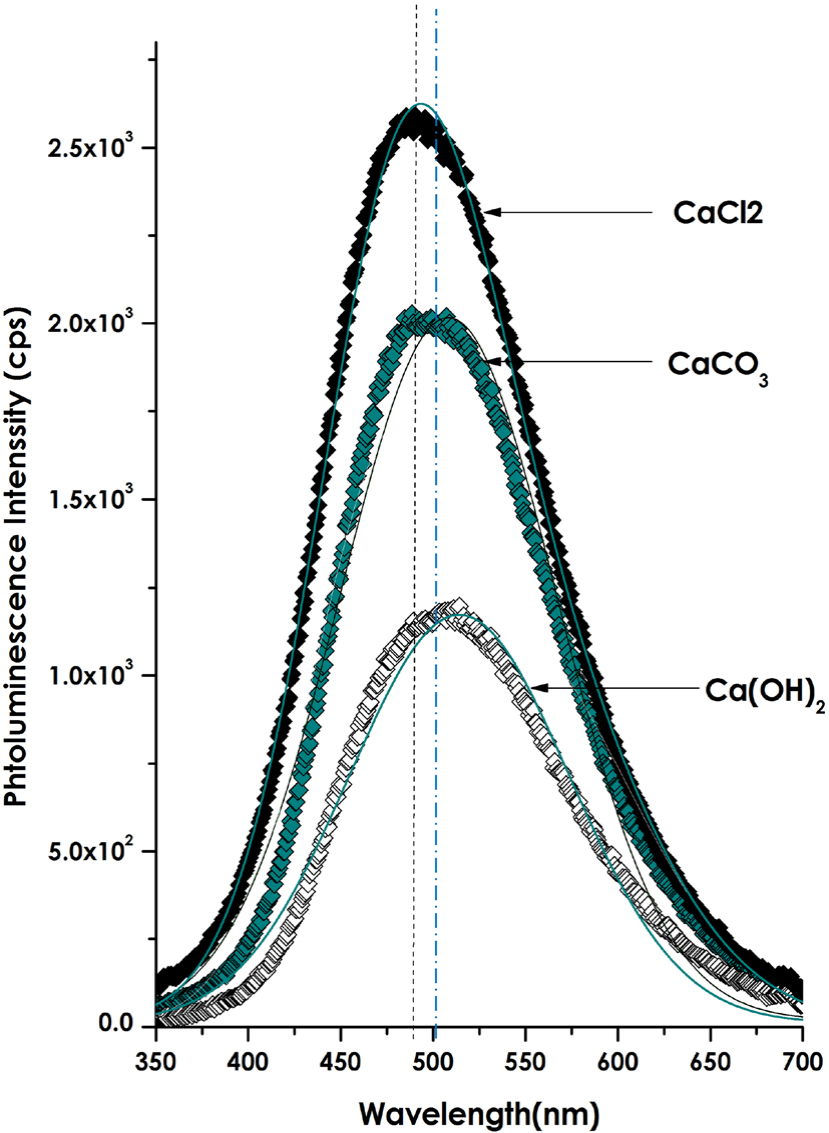
Room temperature photoluminescence of the bio-synthesized nanoscaled CaCO_3_ as well as the intermediary product of Ca(OH)2 as well as the initial precursor CaCl_2_.

Figure S3a reports the spectral emission of the exciting source, the excitation source consists of major emissions centered at 242.7, 263.5, 281.5, and 318.5 nm. Figure S3b displays the mission of the *Hyphaene thebaica* powder used as an effective chelating agent. It consists of a relatively minute emissions centered at 508.2 and 581.5 nm. Figure 6 displays the room temperature photoluminescence of the bio-synthesized nanoscaled CaCO_3_ (well as the intermediary product of Ca(OH)_2_ as well as the initial precursor CaCl_2_ under the external excitation of Fig. S3a. Each and all exhibit a major broad emission centred at about ~ 490.1 nm, ~ 496.9 and ~ 507.2 nm for CaCl_2_, CaCO_3_ and Ca(OH)_2_ respectively. In view of the spectral position similarly of the maximum emission, this later is likely to be of the same physical origin. Such a broad emission of the Bio-engineered CaCO_3_ could be caused by surface or volume Ca defects or recombination of electrons of (Ca^+^-CO_3-_)-centres for the intrinsic emission band^67,68^. Likewise, it can be caused by the intrinsic emission of the electron–hole recombination where a CO^3−^ ion plays the role of the hole and Ca^+^ the role of the electron^69–72^.

### Crystallographic studies

**Figure 7:**
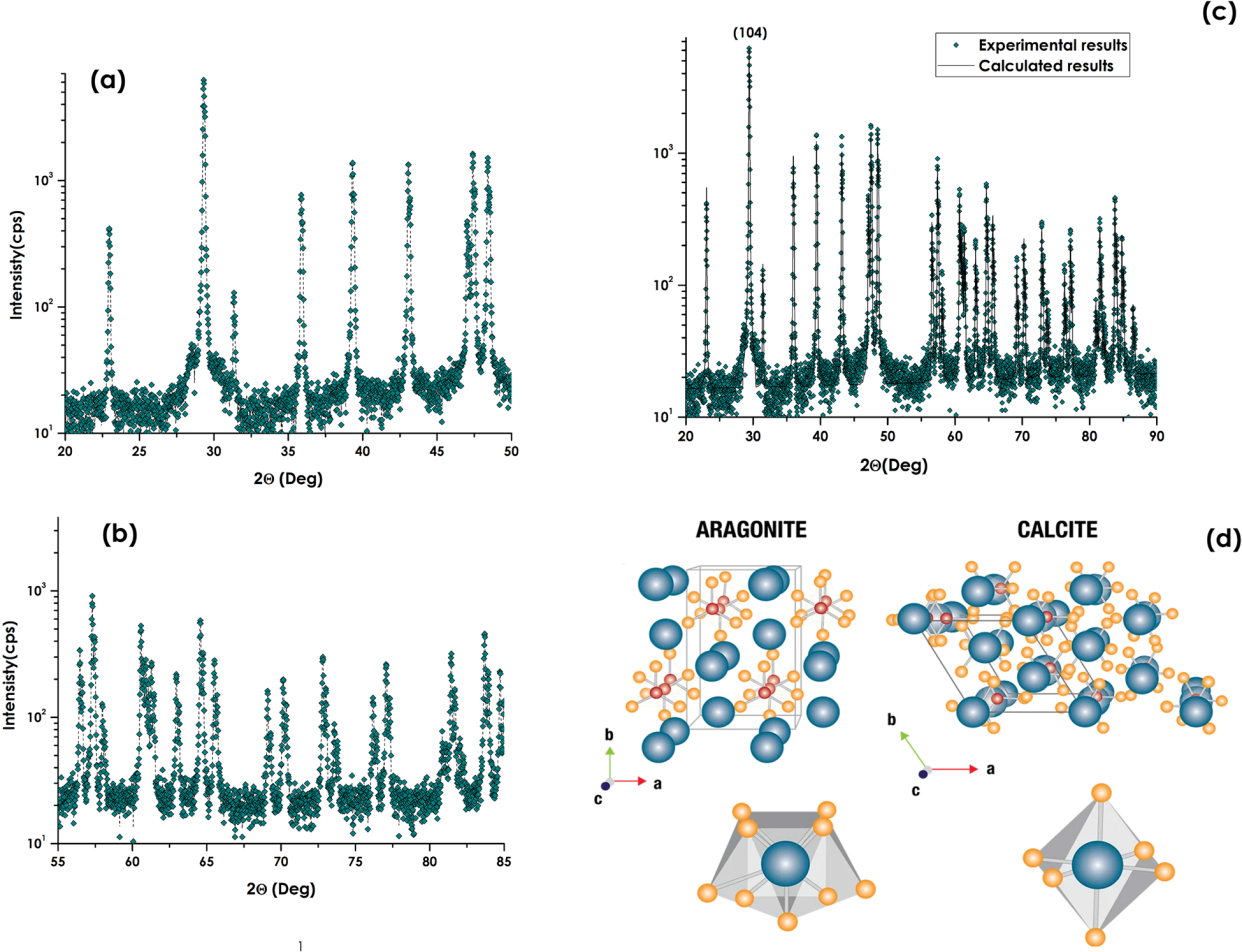
The Θ–2Θ X-rays diffraction spectra within the angular range of (**a**) 20°–50° and (**b**) 55°–85°, (**c**) full XRD profile with the MAUD simulation and (**d**) proposed calcite crystallographic structure of the bio-engineered CaCO_3_ 1-D nanoparticles.

As established, in standard P–T conditions, CaCO_3_ has three polymorphs: calcite (rhombohedral), Aragonite (orthorhombic), and Vaterite (orthorhombic). Calcite is thermodynamically the most stable in most environments^61,65^. Figure 7a,b display the Θ–2Θ X-rays diffraction spectra within the angular range of 20°–50° and 55°–85° respectively. 16 observed Bragg peaks were observed as summarized in Table 1. The most intense is the one centred at 29.45° which is the most intense characteristic (104) reflection of the Calcite so is the case of each and al Bragg diffraction peaks. Following the MAUD treatment. The simulation of the full spectrum (Fig. 7c), the derived lattice parameters are $\left\langle {\text{a}} \right\rangle$ = $\left\langle {\text{b}} \right\rangle$ = 4.883 Å and $\left\langle {\text{c}} \right\rangle$ = 16.310 Å. Within the experimental bar errors, these parameters are in agreement with pure Calcite trigonal crystallographic structure (Fig. 7d) with a space group R-3c^61,73^. One should mention that the lattice parameters of the 3 major phases in their bulk configuration are as follows:

**Table 1:** Angular position of the various Bragg diffraction peaks and their corresponding miller indexation of the bio-engineered CaCO_3_ nanorods.

Angular position of the main Bragg peaks 2Θ (°)	Miller indexation (hki)	Corresponding CaCO_3_ phase
22.95	012	Calcite only
29.45	104	Calcite + aragonite
31.46	006	Calcite only
36.06	110	Calcite only
39.51	113	Calcite only
43.14	202	Calcite only
47.54	018	Calcite only
48.70	116	Calcite only
56.62	211	Calcite only
57.51	112	Calcite only
60.76	214	Calcite only
64.80	300	Calcite only
73.01	128	Calcite only
77.43	220	Calcite only
81.67	214	Calcite only
83.95	134	Calcite only

Bulk Calcite trigonal: $\left\langle {\text{a}} \right\rangle$ = $\left\langle {\text{b}} \right\rangle$ = 4.9892 Å and $\left\langle {\text{c}} \right\rangle$ = 17.06089 Å, Space group R-3c.

Bulk Aragonite orthorhombic: $\left\langle {\text{a}} \right\rangle$ = $\left\langle {\text{b}} \right\rangle$ = 4.9803 Å and $\left\langle {\text{c}} \right\rangle$ = 17.0187 Å.

Bulk Vaterite orthorhombic: $\left\langle {\text{a}} \right\rangle$ = $\left\langle {\text{b}} \right\rangle$ = 4.1226 Å and $\left\langle {\text{c}} \right\rangle$ = 8.4653 À, Space group P6_3_/mmc.

In addition, the derived lattice parameters ($\left\langle {\text{a}} \right\rangle$ = $\left\langle {\text{b}} \right\rangle$ = 4.883 Å, $\left\langle {\text{c}} \right\rangle$ = 16.310 Å) are smaller than their corresponding bulk values, suggesting that the CaCO3 nanorods are under a compressive stress within the 3 crystallographic directions.

### Ethical approval

In view of the usage of the usage of "*Hyphaene thebaica* L. mart fruit” for scientific purpose, a full permission to collect it was allowed and its usage was permitted. Likewise, It is to point to the fact that the usage of the collected “*Hyphaene thebaica* L. mart fruit” complies with UNISA and iThemba LABS-National Research Foundation of South Africa institutional, national, and international guidelines and legislation.

## R&D translations: potential technological applications

### Properties: light scattering and white pigment applications

Figure S4 displays the UV–VIS–NIR optical absorbance of the *Hyphaene thebaica* natural extract, the extract with the CaCl_2_ (just upon dissolution), the extract with the CaCl_2_ (after reaction over 24 h and formation of colloidal Ca(OH)_2_), extract with the CaCl_2_ (after reaction over 24 h and formation of colloidal Ca(OH)_2_ and bubbling with CO_2_). Following the CO_2_ bubbling, the Ca(OH)_2_ colloidal solution becomes turbid which was interpreted as related to the formation of colloidal CaCO_3_. As one can notice in Fig. S4a and its zoom (Fig. S4b, while the various solutions exhibit a broad optical absorbances from 200 to 400 nm, the colloidal CaCO_3_ solution exhibit a relatively sharper absorbance in the deep blue region (200–230 nm) in addition to a constant plateau over 230 nm. The observed behavior is characteristic of pure CaCO_3_’s absorbance^74^. Likewise, such a specific absorbance peak within the 200–230 is in line with the CaCO3’s bandgap of about 5 eV.

To sustain such an hypothesis, the 2 last colloidal solutions (after reaction over 24 h and formation of Ca(OH)2), and (after reaction over 24 h, formation of Ca(OH)2 and bubbling with CO_2_) were centrifuged. Figure S5 display the corresponding UV–VIS–NIR diffuse reflectances. Both spectra are in line with the pure Ca(OH)_2_ and CaCO_3_^75^.

**Figure 8:**
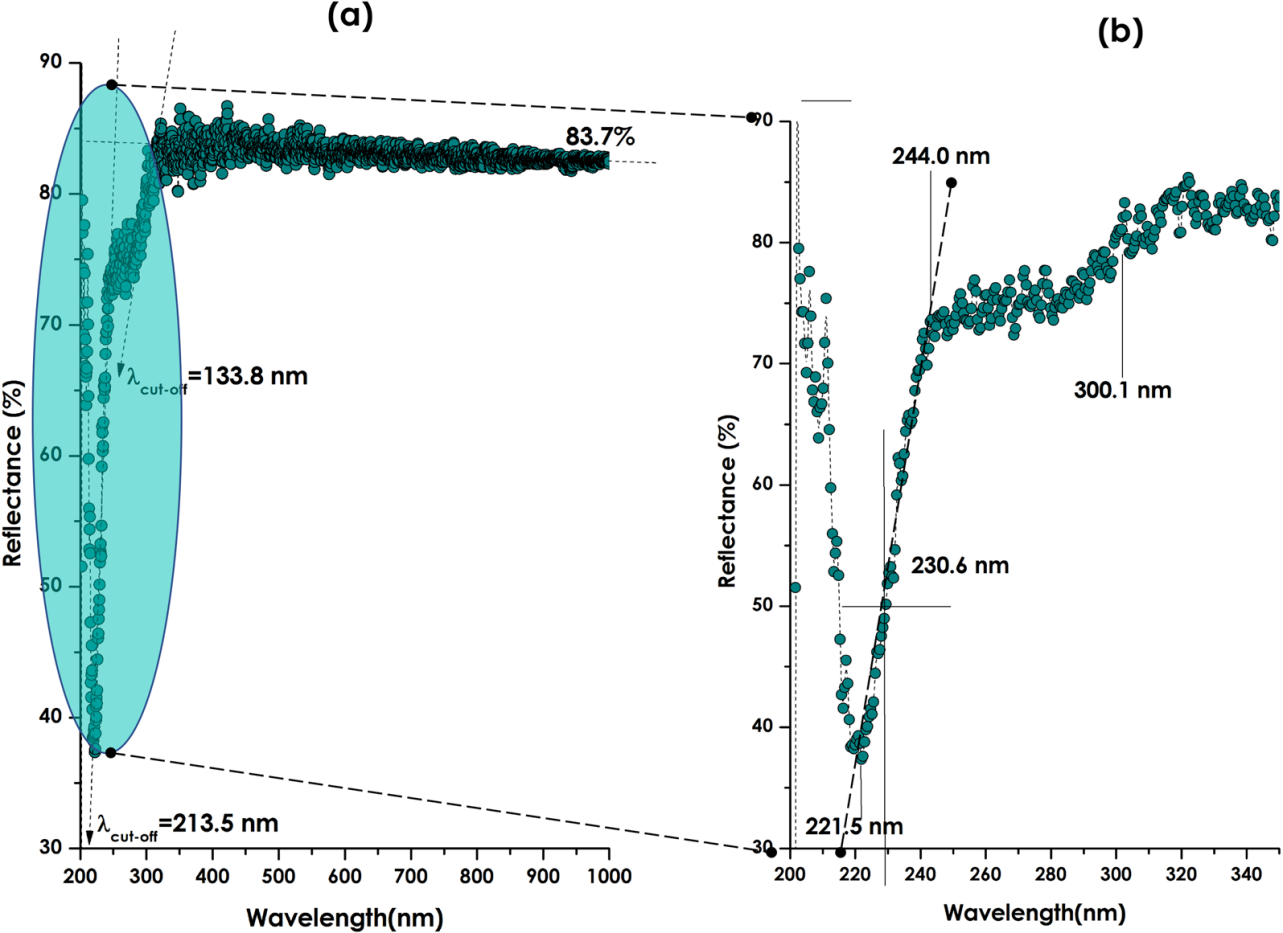
(**a**) Standard diffuse reflectance spectrum under normal incidence of the bio-engineered CaCO_3_ 1-D nanoparticles within the spectral range of 200–1000 nm, (**b**) the corresponding zoom on the UV-Bleu spectral region of 200–345 nm.

Figure 8a displays the standard diffuse reflectance spectrum under normal incidence of the bio-engineered CaCO_3_ nanoparticles (in their pelletised powder form) within the spectral range of 200–1000 nm. Figure 8b displays the corresponding zoom on the UV-Bleu spectral region of 200–345 nm. From Fig. 8a, one can safely state that there are 2 major regions, separated approximately at 300.1 nm, above which the average diffuse reflectivity is about ~ 83.7%. Such an elevated reflectivity within the visible (VIS) and Near infrared (NIR) solar spectral regions is a characteristic of highly reflecting solar materials equivalent to that of standard white pigments including BaSO_4_, ZnO and TiO_2_^76^. Henceforth, one could safely conclude that the bio-engineered CaCO_3_ nanoparticles could be a potential compound for white pigment coatings’ applications.

If one considers the zoom of Fig. 8b, one could single out the minimum in the diffuse reflectance centred approximately at about ~ 221.5 nm with a reflectivity as low as 37.5% and the region delimited between 244.0 nm and 301.1 nm. If one consider the cut-off wavelengths λ_cut-off_ as defined as the limit between the low and high reflectivity regions, then the value of λ_cut-off_ could be approximated as defined by the intersection of the slope with the wavelength x-axis as indicated in Fig. 8a (dashed line intersecting the x-axis). Such an intersection is ~ 213.5 nm. If so, one could associate an optical bandgap of E_g_ = hc/(λ_cut-off_ = 6.42 eV (λ_cut-off_ ~ 213.5 nm) (h, Plank’s constant and c, Celerity of light). This latter value of E_g_ = 6.42 eV is not far from the experimental bulk Calcite value of 6.0 ± 0.35 eV^77,78^.

### Properties: nanofertilizing response

As in the case of Zinc, Calcium is a crucial plant nutrient playing a vital role in maintaining plant cellular metabolism. As a biocatalyst becoming functional through Calcium ionic species, These Calcium ionic species are concerned with hydrocarbons metabolism, maintenance of cellular membranes, leaf morphology, physiology of membrane, protein production,…^79^. Likewise, it is established that Ca^2+^ activates various types of enzymes, hence is a pivotal co-factor within the plant system. More precisely, It participates in membrane transport metabolisms, nitrate take-up and in biomass proportion^79^ and photosynthetic rate^80–82^. In addition, it has been demonstrated that Ca^2+^ enhances the saltiness and improves the plant development^83–85^. Calcium is found in upwards of 80 compounds some of the time called calcium salts, primarily, calcium carbonate. CaCO_3_ is an essential part of the nursery lime, otherwise called agrarian lime. In addition, holding limit of acidic soils. CaCO_3_ sources, for example, limestone and chalk, alongside other synthetic compounds are utilized in the readiness of agrarian lime.

In view of investigating the effectiveness of the currently bio-engineered CaCO_3_ nanoparticles, they were tested as a bio/nano-fertilizer in the case of *Lycopersicum esculentum* (Tomato). In this regard, a similar study with the same parameters and growth conditions was performed as the one conducted previously with ZnO nanoparticles^68^. For the current study, 3 solutions of the nano-scaled CaCO_3_ colloidal solutions were prepared at various concentrations (0.01, 0.03 and 0.05 g/l) with a reference plant as the control, 3 other *Lycopersicum esculentum* plants were considered. These latter were fed regularly with the colloidal solutions while the control one was fed with pure water only.

Accordingly, the average plant’s height, the average number of leaves as well as the average number of days to flowering were collected. Table 2 summarizes such a set of results.

**Table 2:** Evolution of the Lycopersicum *esculentum* plants’ versus the CaCO_3_ nutrient concentration and the control sample.

CaCO_3_ concentration (g/l)	Average plant’s height (± 5 mm)	Average number of leaves (± 20)	Average number of days to flowering
Control	23.1	68	118
0.01	32.7	157	94
0.03	34.1	103	104
0.05	27.2	79	108

**Figure 9:**
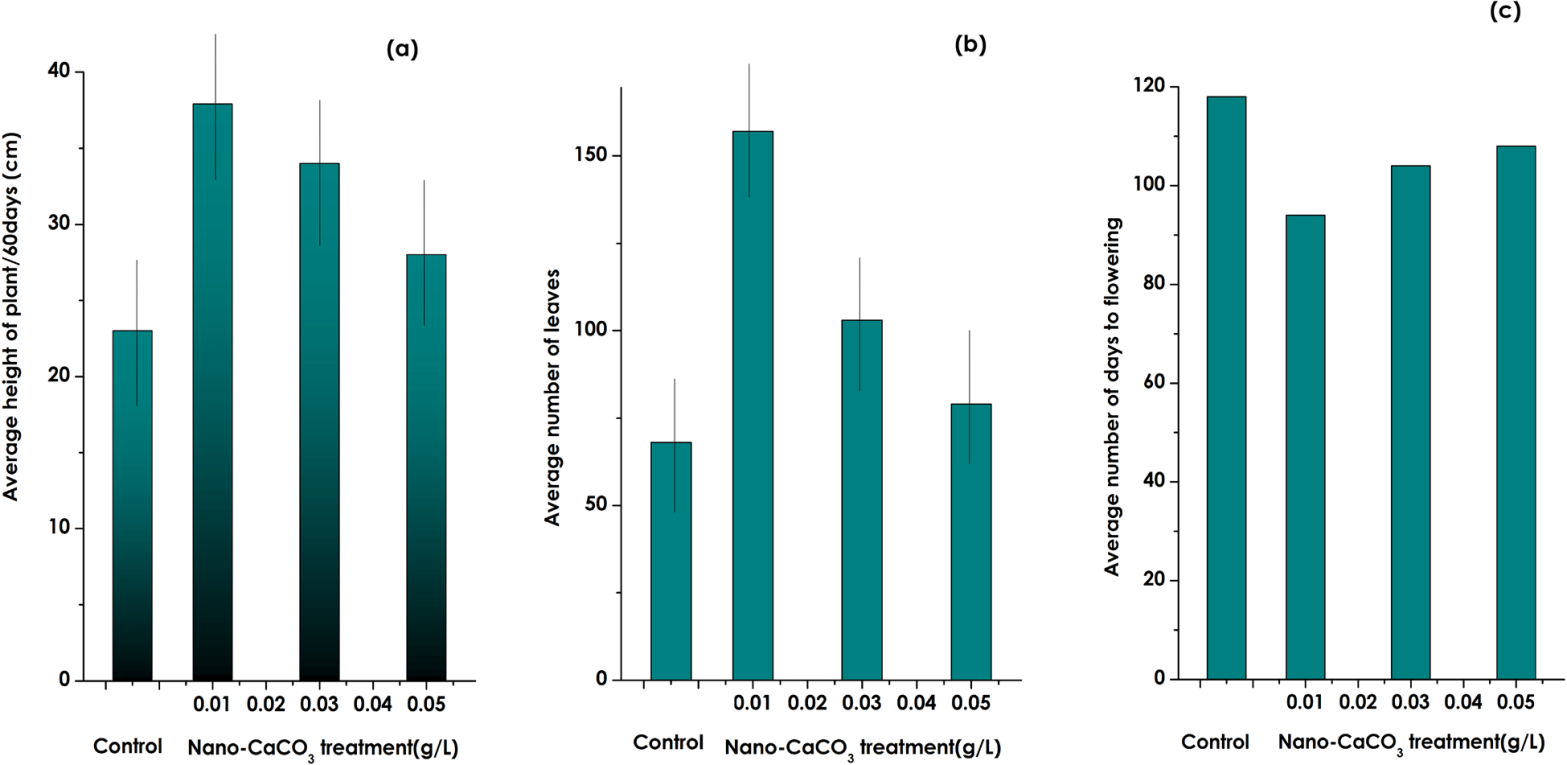
Evolution versus the bio-engineered CaCO_3_ 1-D nanoparticles. Nutrient concentration of (**a**) the average of plant’s height, (**b**) the average number of leaves and (**c**) the average days to flowering relatively to the control sample.

Figure 9a displays the evolution of the average of plant’s height versus the CaCO_3_ nutrient concentration. As one can, crystal clearly observe that the plant’s height is higher than that of the control one for each of the CaCO_3_ nutrient’s concentration especially for the lowest value of 0.01 g/l. A similar behaviour is observed for the average number of leaves vs nutrient concentration (Fig. 9b). Figure 9c seems to be of a special interest. It indicates that the average days to flowering is lower with the CaCO_3_ nutrient concentration relatively to the control sample. Especially for the 0.01 g/l, the flowering is 24 days before the control. As a pre-conclusion, the plant’s growth parameters are far competitive relatively to the control especially for the lowest concentration of 0.01 g/l which seems the optimal value within this conditions of experimentation.

### Properties: cement binder applications

Within the building sector, especially via cement within which CaCO_3_ is a pivotal chemical component, it is established that CaCO_3_ contributes increasing the concrete’s strength and its workability^86–89^. It also improves the concrete’s particle packing while providing concrete with a spacer effect, and promotes self-compacting properties of concrete^90–92^. In addition, CaCO_3_ reduces porosity and air void in concrete and adds to smoother surfaces^87^. From cost effectiveness viewpoint, CaCO_3_ can be used as a filler in Portland cement, reducing the product’s high cost. It was evidenced that replacement of cement by just 2% CaCO_3_ helps reduce the CO_2_ emission from cement plants by 69%, meeting the economic and environmental aspects of fly ash as well as contributing equal positive effects to concrete^93–95^.

**Figure 10:**
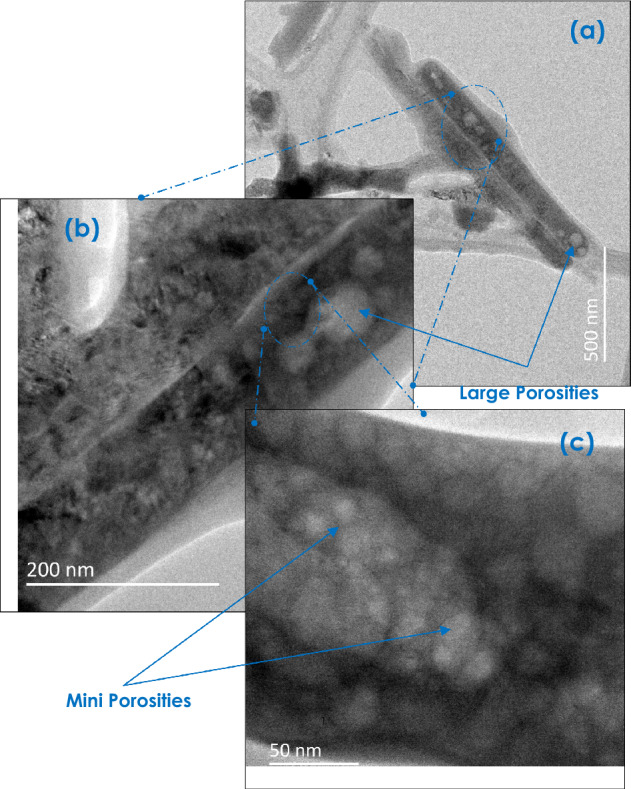
Multi-scale porosity in the bio-engineered CaCO_3_ 1-D nanoparticles.

As per Fig. 1, the bio-engineered CaCO_3_ is nano-scaled in size, and hence has a finer particles size as compared to the Ordinary Portland Cement (OPC) particles (which is in the range of 10μm in average)^95^. This fine aspect would likely improve the particle packing of concrete and give a superior spacer effect. Also, the concrete with CaCO_3_ replacement possess a higher slump, which increases the workability. In addition, in a statistical spatial distribution, the 1-D morphology of the CaCO_3_ nanoparticles would favour if not enhance the local mechanical strength of the CaCO_3_/Cement composite as a local reinforcer. Last but not least, the porosity of the CaCO_3_ nanorods (Fig. 10) could, a priori, favour an enhanced binding in view of the high surface to volume of the individual porous CaCO_3_ nanorods.

Also, as it was mentioned previously (Sect. "Thermogravimetry analysis"), compared to literature, the general TGA and DSC variations/trends of the bio-engineered CaCO_3_ are, equivalent to that of bulk CaCO_3_ but with a significant shift to lower temperatures. More accurately, the decomposition and phase transition temperature are ~ 648.8 °C instead of ~ 750 °C for Bulk. This is likely due to the high surface to volume ratio of the current nanoscaled CaCO_3_ comparatively to their bulk equivalent^96,97^. This would improve its workability within the cement composite.

In view of sustaining the above mentioned cement applications, a mixture of the CaCO_3_ nano-rods with standard Ordinary Portland Cement (OPC) particles will be performed. Their mechanical and thermogravimetric properties will be investigated accordingly including early degradation in harsh environments.

One should highlight that Aragonite needle-like particles were synthesized by bubbling CO_2_-containing gas into Ca(OH)_2_ aqueous slurry in the presence of a significant amount of MgCl_2_^98^. It was elucidated that calcite needle-like particles can be fabricated by calcining aragonite needle-like particles at 500 °C. This fabrication method of thermodynamically stable calcite needle-like particles may broaden the applications of calcium carbonate as a filling material.

It is to been emphasized that similar green approaches have been validated previously by Wang et al.^99^, Nakayama et al.^100^, Babou-Kammoe et al.^101^ as well as Watanabe et al.^102^. More precisely, Wang et al. have used Fucoidan-mediated compound with Sodium carbonate in addition to CaCl2 mixed with a fucoidan solutions. In the case of Nakayama et al., amorphous calcium carbonate, CaCl_2_ aqueous solution with PAA and Na_2_CO_3_ aqueous solution. In the case of Babou-Kammoe et al., several starting materials were used including Sodium carbonate (Na_2_CO_3_), sodium hydroxide (NaOH) and calcium nitrate tetrahydrate (Ca(NO_3_)_2_, 4H_2_O). Watanabe et al. have used also several precursors such as Calcium chloride powder and ammonium aqueous solution. While, the above methodologies have their merit as potentials ways, the require a relatively larger set of precursors while the proposed one requires H_2_O as the universal solvent and CaCl_2_ as the sole precursor. A priori, this later can be any Ca salt. Likewise, and as it would be reported soon, this presented room temperature green approach.

## Conclusions

This study validated the possibility of green bio-engineering of 1-D single phase calcite CaCO_3_ porous nano-rods. The bio-synthesis is performed at room temperature with H_2_O as universal solvent with CaCl_2_ and CO_2_ as the unique source of Ca and CO_3_. The bio-engineered nano-scaled CaCO_3_ exhibited an effective multi-functionality including a significant efficiency as white pigment, an effective response as a nano-fertilizer and a potential source for green cement and cement industry. The same approach is likely valid for the synthesis of various Carbonates including but not limited to, FeCO_3_, CuCO_3_, MgCO_3_, NiCuO_3_, SrCuO_3_,… Yet the natural extract of *Hyphaene thebaica* L. Mart fruit was used as an effective chelating agent at room temperature, it is safe to confirm that natural extract of other plants with equivalent phyto/enzymes composition would also be effective for the bio-synthesis of Carbonates.

### Supplementary Information

Supplementary Figures.

## Data Availability

The datasets used and/or analysed during the current study available from the corresponding author on reasonable request.
