# Supplementary material for: Room temperature bio-engineered multifunctional carbonates for CO2 sequestration and valorization
Source: Sci Rep. 2023 Oct 5;13:16783. doi: 10.1038/s41598-023-42905-5 (PMC10556044; doi:10.1038/s41598-023-42905-5)
Supplement: Supplementary file 1 — Supplementary Figures. [file 41598_2023_42905_MOESM1_ESM.pdf]

Room Temperature Bio-Engineered Multifunctional Carbonates for CO_2 Sequestration &Valorization

H. Mohamed[1-2], K. Hkiri[1-2], N. Botha[1-2], K. Cloete[1-2] , Sh. Azizi[1-2,] , A.A.Q. Ahmed[1-2,] , R. Morad[1-2], Th. Motlamane[1-2], A. Krief [1-2,3],A. Gibaud[1-2,4], M. Henini [1-2,5] ,M. Chaker [1-2,6] , I. Ahmad [1-2,7], M. Maaza[1-2*],

[1]*UNESCO-UNISA Africa Chair in Nanosciences-Nanotechnology, College of Graduate Studies, University of South Africa, Muckleneuk ridge, PO Box 392, Pretoria, South Africa*

[2]*Nanosciences African Network (NANOAFNET), Materials Research Dept., iThemba LABS-National Research Foundation of South Africa, 1 Old Faure Road, Somerset West, Western Cape 7129, PO Box 722, South Africa*

[3] *Chem. Dept (CMI laboratory), University of Namur, 2 rue Joseph Grafé, B-5000, Namur-Belgium*

[4]*IMMM, UMR 6283 CNRS , Bd O. Messiaen, 72085 Le Mans cedex 09, University of Le Maine, Le Mans-France*

[5] *Physics & Astronomy Dept., Nottingham University, NG7 2RD7, UK*

[6] *INRS-Energie et matériaux, 1650 Lionel-Boulet, Varennes, Québec J3X 1S2 Canada*

[7]*Experimental Physics Directorate (EPD), National Center for Physics, Islamabad 44000, Pakistan*

Corresponding author*: M. Maaza: Maaza@tlabs.ac.za, Maazam@unisa.ac.za

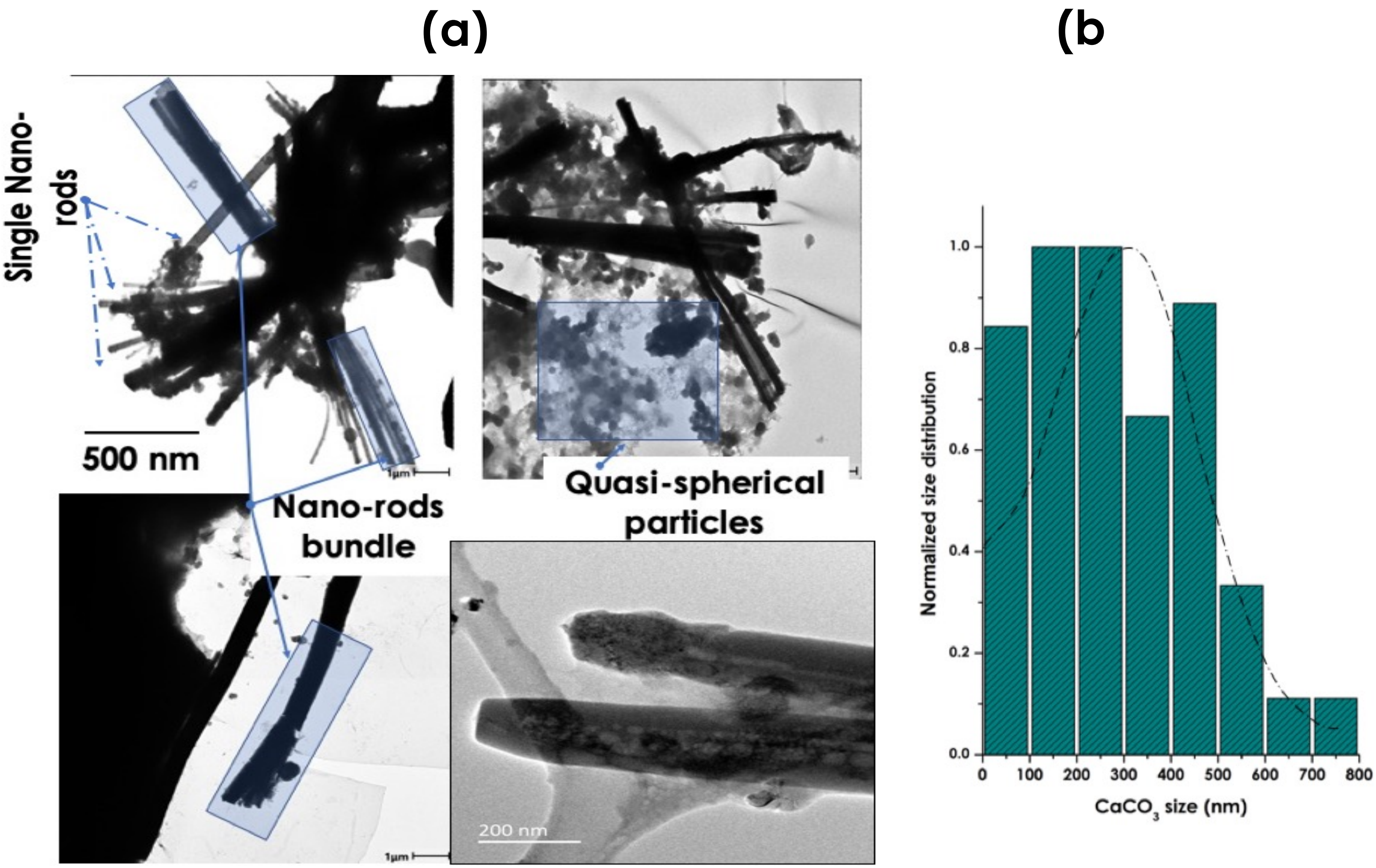

Fig.S.1.: (a) Low magnification Transmission electron microscopy of the $CaCO_3$ rods, (b) their normalized basal size distribution.

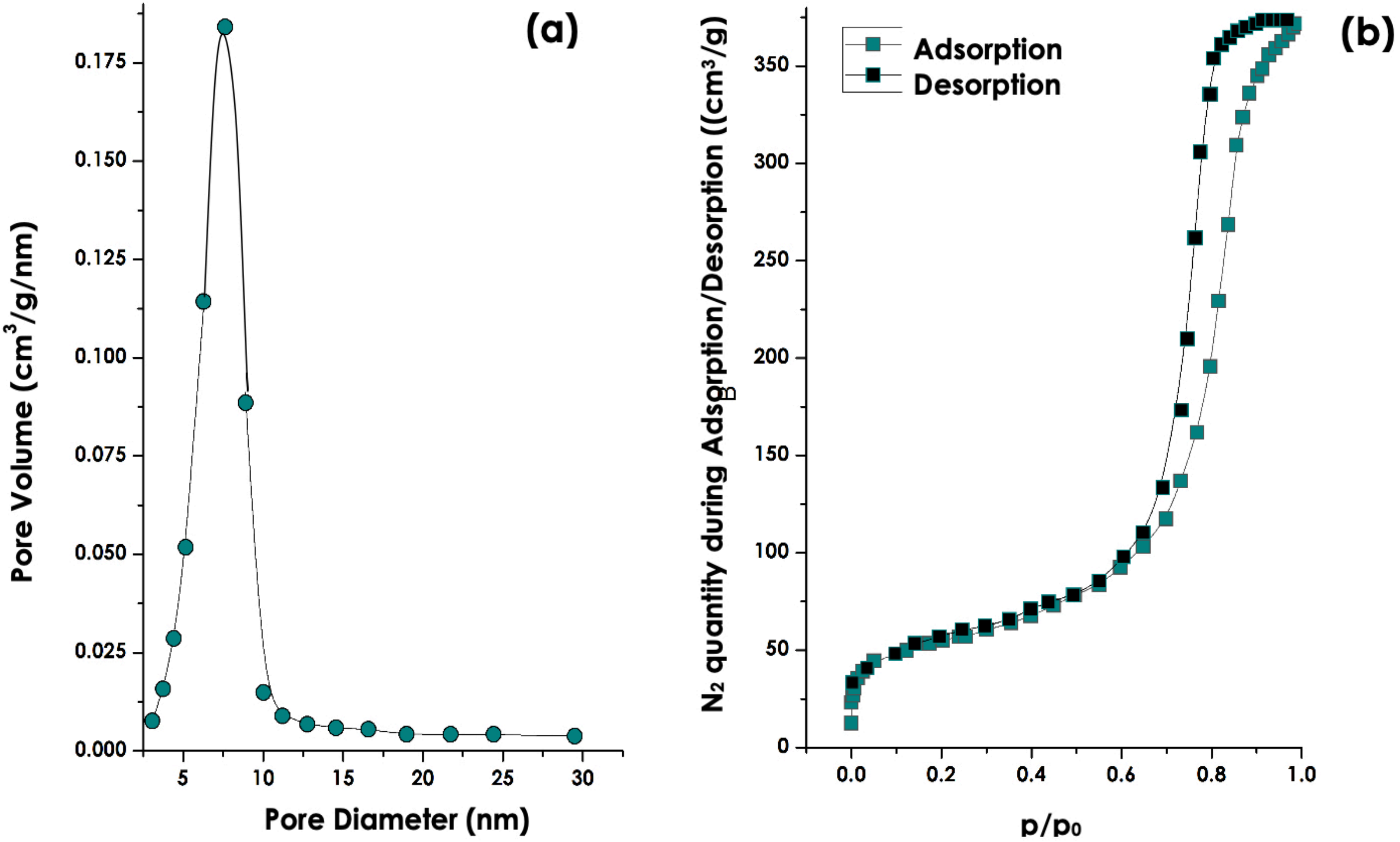

Fig.S.2.: (a) BET porosity distribution of the $CaCO_3$ rods, (b)their corresponding N_2 adsorption/ desorption

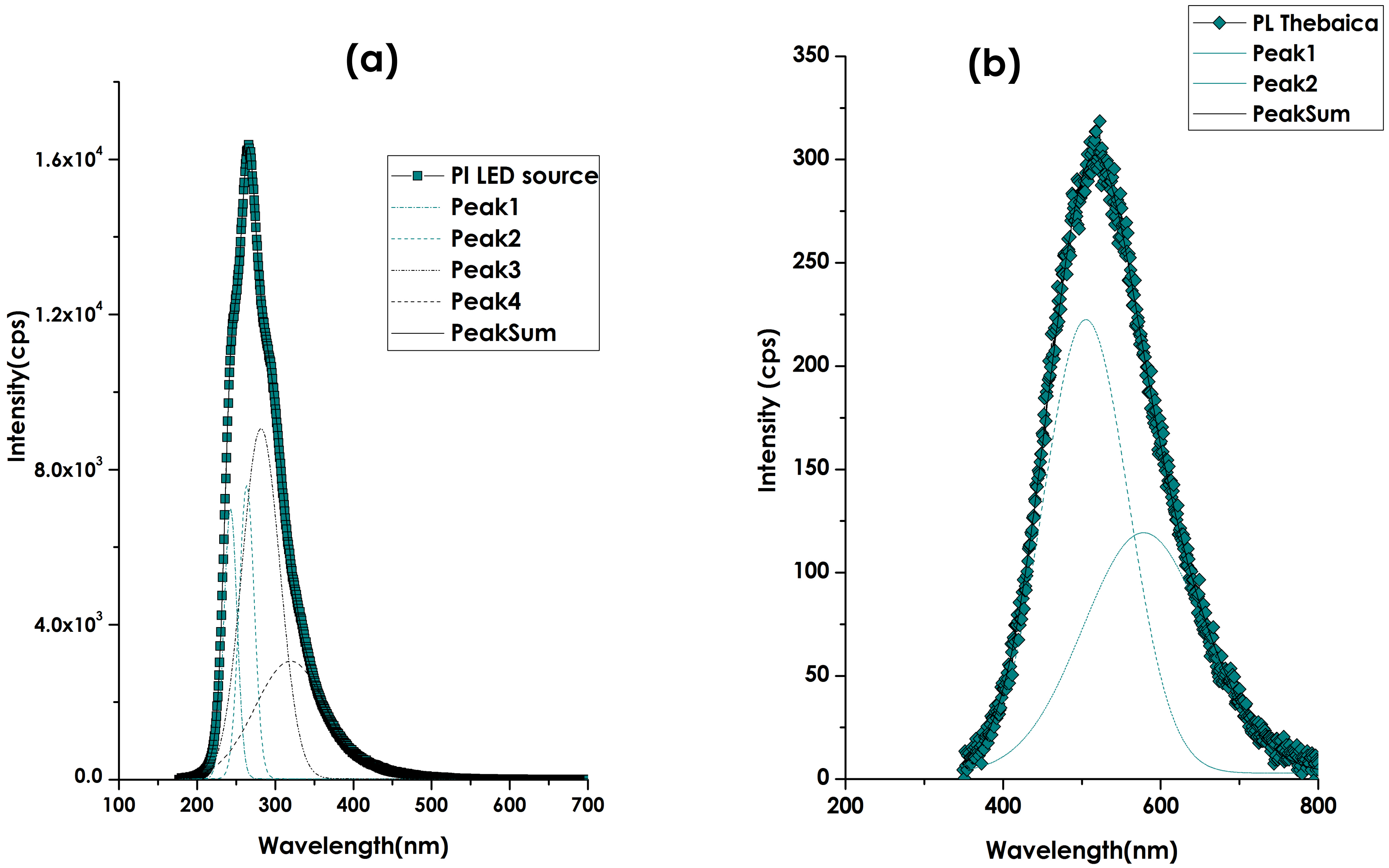

Fig.S.3.: (a) Spectral emission of the exciting source, (b) Emission of the thebaica hypaene powder used as an effective chelating agent.

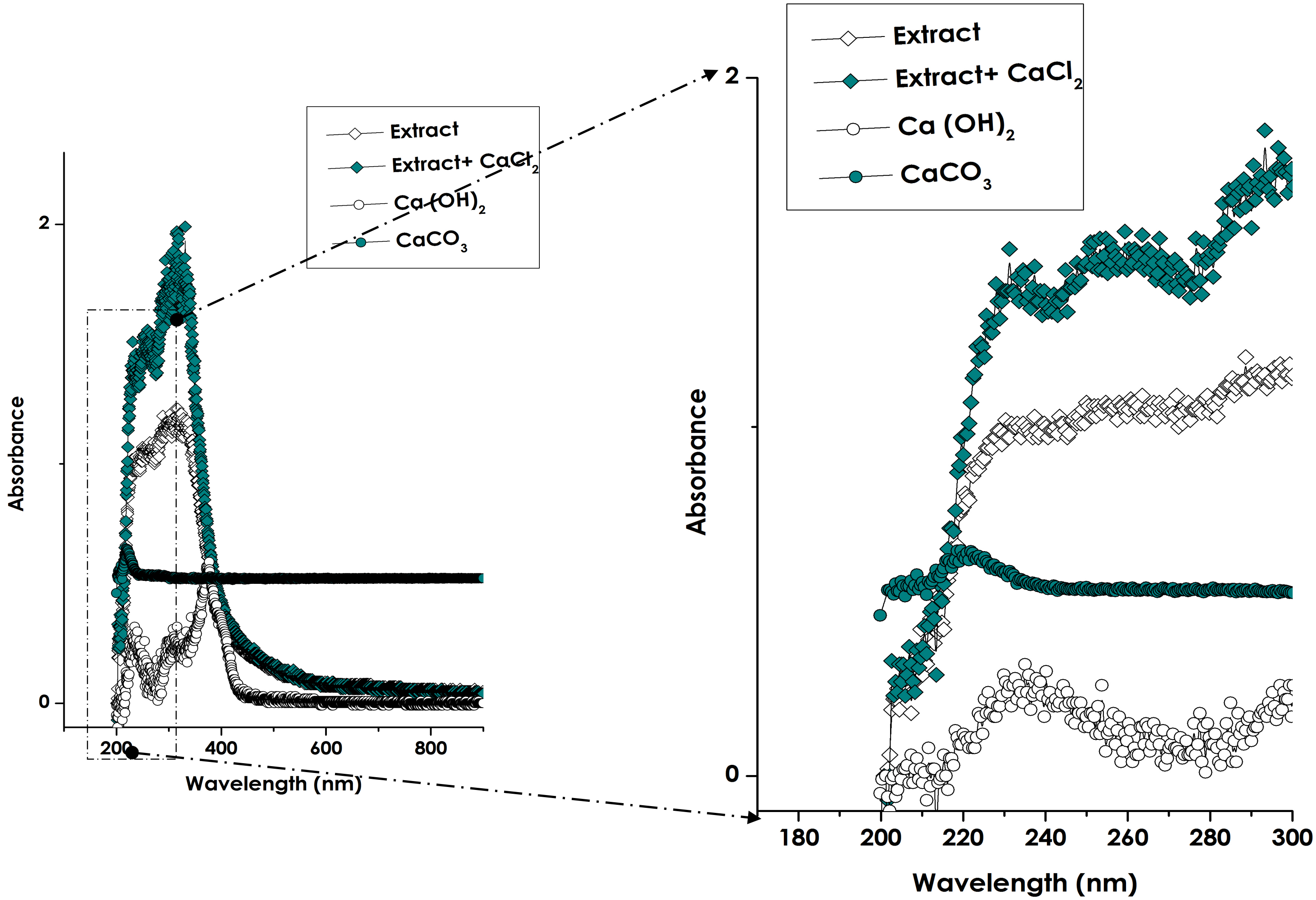

Fig.S.4.: UV-VIS-NIR optical absorbance of the thebaica hypaene natural extract , extract with the $CaCl_2$ (just upon dissolution), extract with the $CaCl_2$ (after reaction over 24h & formation of colloidal $Ca(OH)_2$), extract with the $CaCl_2$ (after reaction over 24h & formation of colloidal $Ca(OH)_2$ & bubbling with CO_2),

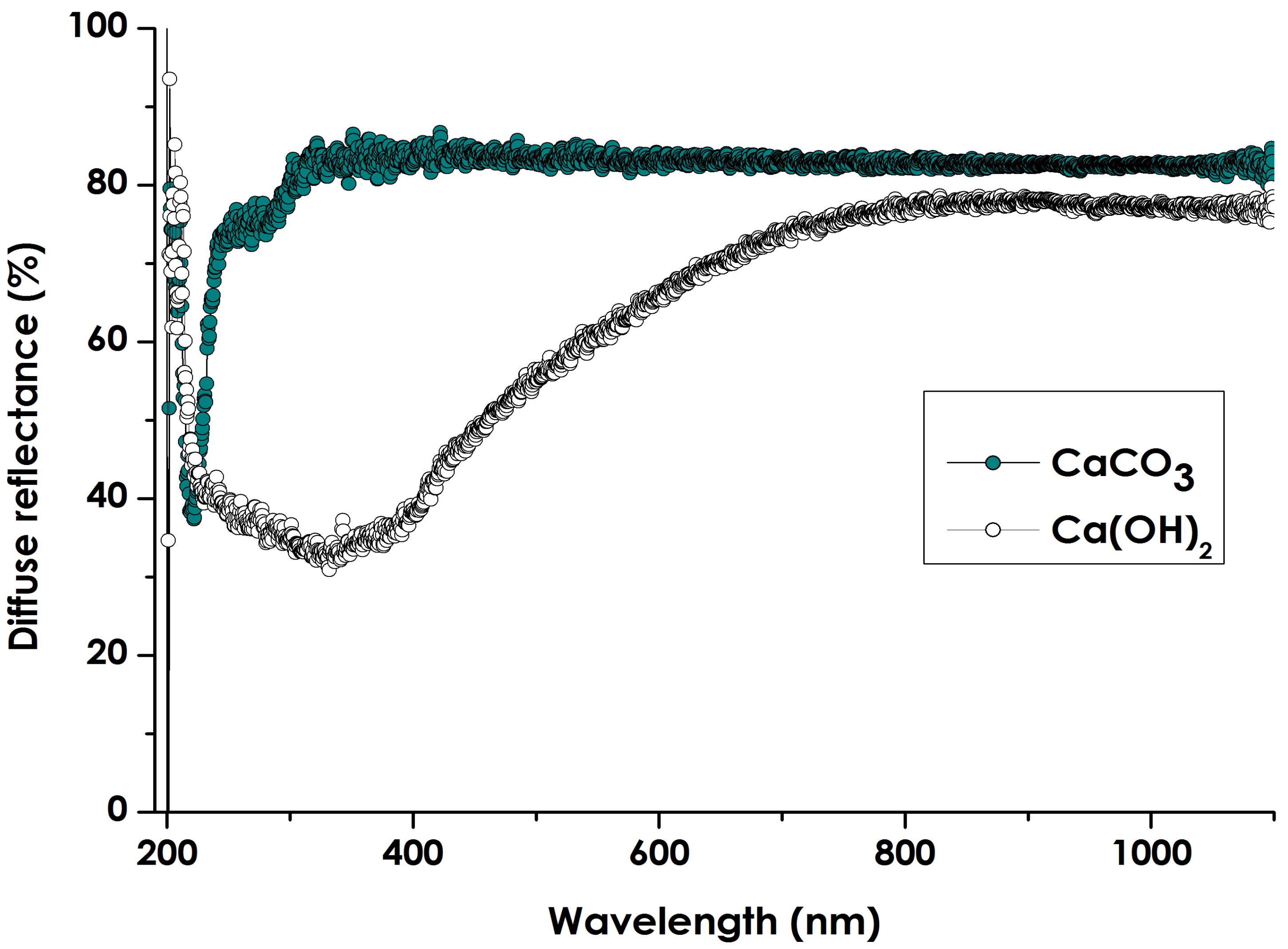

Fig.S.5.: UV-VIS-NIR diffuse reflectance of the $Ca(OH)_2$ & $CaCO_3$ obtained by centrifugation of the colloidal suspensions obtained (after reaction over 24h & formation of $Ca(OH)_2$), and (after reaction over 24h, formation of $Ca(OH)_2$ & bubbling with CO_2),
